# Detection of Volatile Organic Compounds by Weight-Detectable Sensors coated with Metal-Organic Frameworks

**DOI:** 10.1038/srep06247

**Published:** 2014-09-01

**Authors:** Hiroki Yamagiwa, Seiko Sato, Tadashi Fukawa, Tsuyoshi Ikehara, Ryutaro Maeda, Takashi Mihara, Mutsumi Kimura

**Affiliations:** 1 Faculty of Textile Science and Technology, Shinshu University, Ueda 386-8567, Japan; 2 National Institute of Advanced Industrial Science and Technology (AIST), 1-2 Namiki, Tsukuba 305-8564, Japan; 3 Future Creation Laboratory, Olympus Corporation, Tokyo 192-8512, Japan

## Abstract

Detection of volatile organic compounds (VOCs) using weight-detectable quartz microbalance and silicon-based microcantilever sensors coated with crystalline metal-organic framework (MOF) thin films is described in this paper. The thin films of two MOFs were grown from COOH-terminated self-assembled monolayers onto the gold electrodes of sensor platforms. The MOF layers worked as the effective concentrators of VOC gases, and the adsorption/desorption processes of the VOCs could be monitored by the frequency changes of weight-detectable sensors. Moreover, the MOF layers provided VOC sensing selectivity to the weight-detectable sensors through the size-selective adsorption of the VOCs within the regulated nanospace of the MOFs.

On-site and real-time detection of VOCs is a critical and challenging issue for environmental and health monitoring. VOC detection systems have been developed as electronic nose systems composed of an array of several different sensors and a pattern recognition system^1^,^2^,^3^. Electronic gas sensors within the electronic nose systems are capable of converting chemical information into an output signal, and each sensor in the array can generate different signals in response to the concentration and specific interaction with the target VOCs^4^. Gas sensors, which are based on the chemical sensitivity of metal oxide semiconductors (MOS), are commercially readily available^5^. Although MOS sensors have been widely used to create arrays for VOC sensing, these sensors require heating the sensing layer at 200–400°C for their operation. Electromechanical devices such as quartz crystal microbalances (QCMs) and microcantilevers have been used in chemical sensing using various sensing materials to overcome these disadvantages of MOS sensors^6^,^7^. These devices detect analytes by sensing small changes in the frequency of a resonant vibration as a result of weight changes on a nanogram level. The sensing materials on electromechanical devices accumulate the target analytes and transduce the weight changes into the frequency shifts of the sensors. We previously demonstrated the detection of VOCs by using the QCM and microcantilever sensor arrays deposited with polymers and organic-inorganic hybrid materials^8^,^9^,^10^,^11^. The large surface area and porosity of porous titanium oxide (TiO_2_) films enhanced their sensitivity to VOC gases^11^. Covering the TiO_2_ surface with polymer layers improved the repeatability and responsibility for the VOC sensing compared to the sensors with only polymer layers. The tunability and structural diversity of organic-inorganic hybrid materials enables for the creation of a pattern recognition library of chemical sensor arrays.

Special attention has been paid to MOFs due to their potential applications such as gas storage/purification, catalysis, and chemosensors based on their tunable nanoporosity and high surface area^12^,^13^,^14^,^15^,^16^,^17^. The connection of metal ions or clusters with multitopic organic linkers creates a regulated nanospace within the extended crystalline structures. The guest molecules can be incorporated into the nanospace of MOFs through their molecular sieving effects, π-π interaction, hydrogen bonding, and electrostatic interactions, etc. The nanospace in MOFs can recognize the size and shape of guest molecules, and their high surface-areas make them promising candidates for a variety of sensing applications. Several groups have reported on chemical detections of small guest molecules though the integration of MOFs into devices such as QCM, surface plasmon resonance spectroscopy, surface acoustic wave devices, and microcantilevers^18^,^19^,^20^. Biemmi et al. demonstrated the direct growth of MOFs on gold electrodes on QCMs to evaluate the water-sorption properties of MOF thin films^21^. Allendorf et al. succeeded in the chemical detection of gases by monitoring the stress generation between a microcantilver and a MOF thin film in response to the molecular adsorption within crystalline MOFs^22^. MOF thin films deposited onto these sensor platforms are an effective concentrator for low-concentration analytes, and also provide the selectivity for analyte species^23^.

We discussed the highly sensitive and selective detection of VOCs through the integration of MOF thin films with weight detectable sensor platforms in this paper. Thin films of two MOFs [Cu_3_(BTC)_2_(H_2_O)_3_]·xH_2_O (BTC = 1,3,5-benzenetricarboxylate)^24^ and [Zn_4_O(BDC)_3_] (BDC = 1,4-benzenedicarboxylate)^25^ were grown from COOH-terminated self-assembled monolayers (SAM) onto the gold electrodes of QCMs or silicon microcantilevers. The structures and morphologies of the resulting MOF films were probed using grazing incidence X-ray diffraction (GIXRD), infrared reflection absorption spectroscopy (FT-IR-RAS), and scanning electron microscopes (SEM) images. The stiff MOF architectures could transduce the weight changes of the VOCs adsorption/desorption into the frequency changes of the weight-detectable sensors, and the adsorption dynamics of the VOCs into the MOFs were monitored directly through the frequency changes of the sensors. The structural difference between Cu_3_(BTC)_2_ and Zn_4_O(BDC)_3_ affected the responsibility and selectivity of the VOC sensing.

## Results

VOC sensors have been developed by modifying the surface of sensor platforms with various kinds of sensing materials, and these sensors can be used for monitoring dynamic processes such as chemisorption, reaction, and intermolecular interactions^1^,^2^,^3^. When the target molecules are captured within the sensing layers on the weight-detectable devices, the sorption amounts can be monitored based on their frequency changes. The VOC sorption and desorption processes within the porous MOFs in this study were monitored by the frequency changes of the surface-modified weight detectable sensors. MOFs can be grown as crystalline thin films on solid surfaces through two self-assembling processes^26^,^27^,^28^,^29^,^30^,^31^. Shekhah et al. reported on the step-by-step synthesis of MOF thin films on a SAM by using two solutions of metal ions and ligands^26^. Bein et al. demonstrated the oriented crystal growth of Cu_3_(BTC)_2_ on different functionalized SAMs using crystallization solutions containing colloidal or molecular building blocks of Cu_3_(BTC)_2_^27^. The film growths on COOH- and OH-terminated SAMs resulted in different crystalline orientations along the [100] and [111] directions. We fabricated MOF thin films on weight-detectable sensors modified with the COOH-terminated SAMs by using crystallization solutions of Cu_3_(BTC)_2_ and Zn_4_O(BDC)_3_.

**Figure 1:**
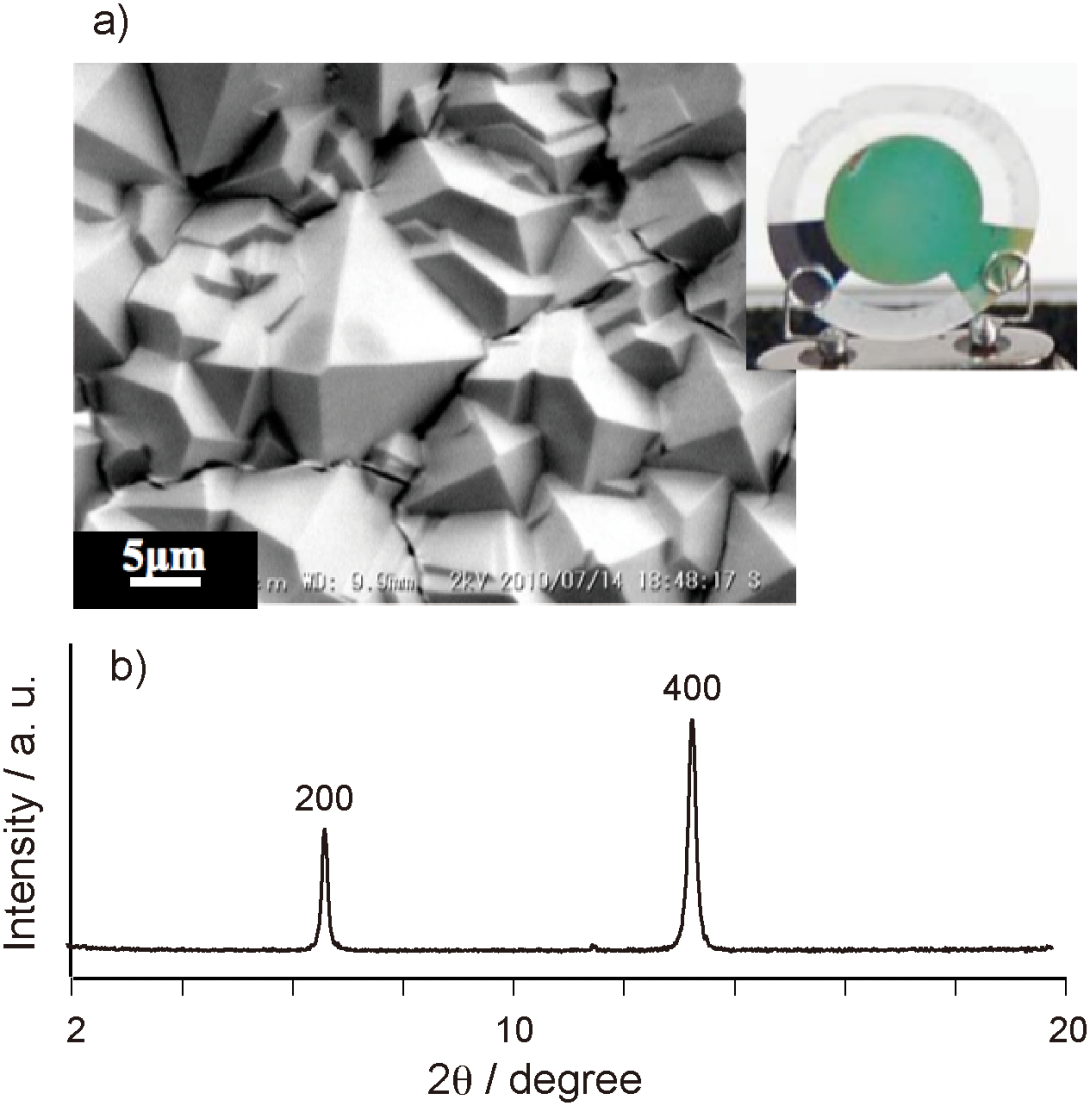
a) SEM image and photograph of Cu_3_(BTC)_2_ thin film grown from COOH-terminated SAM on gold electrode of QCM. b) Out-of-plane XRD data for Cu_3_(BTC)_2_ film on QCM.

The gold electrodes of 9 MHz AT-cut QCMs were functionalized with the SAM monolayer with COOH-terminates. After the formation of the SAM, the QCMs were placed horizontally onto a Teflon plate with a circular window and the gold electrodes were exposed to a clear crystallization solution of Cu_3_(BTC)_2_. While no Cu_3_(BTC)_2_ crystals were obtained on the QCMs without the SAM modification, the dense layer of micrometer-sized Cu_3_(BTC)_2_ crystals on the COOH-terminated SAM was formed with a uniform orientation (Figure 1a). The FT-IR-RAS spectrum of the Cu_3_(BTC)_2_ crystal on the QCM displayed two peaks at 1390 and 1600 cm^−1^, indicating the formation of coordination bonds between the carboxyl groups in the BTC ligand and copper ions^27^ (Fig. S1). A thermo-gravimetric analysis (TGA) of the crystals showed the weight losses of −18.5% from 20 to 120°C (−H_2_O) and −28.4% from 250 to 400°C (−CO_2_ and others)^24^ (Fig. S2). The TGA profile is almost coincidence to the reported profile of Cu_3_(BTC)_2_. The x-ray diffraction pattern of the thin Cu_3_(BTC)_2_ films on the QCMs indicated only two (200) and (400) reflections, implying the highly oriented crystalline growth along the [100] direction on the COOH-terminated SAM layer (Figure 1b, Fig. S3)^27^. The average film thickness of Cu_3_(BTC)_2_ on the QCMs was 500 ± 50 nm estimated from the frequency decrease of the QCMs after the deposition of the crystals, the area of electrode, and the reported density of Cu_3_(BTC)_2_. From these results, highly ordered thin Cu_3_(BTC)_2_ films were formed on the QCM sensors modified with the COOH-terminated SAMs.

The resonant vibration in a QCM resonator is generated in a direction perpendicular to the surface of the quartz crystal by applying a voltage. The generated vibration propagates within the sensing layer, and the QCMs modified with sensing layers detect the adsorption of analytes by sensing the frequency changes of a resonant vibration. The dissipative loss of the resonance propagation in the sensing layer leads to unstable oscillation due to the contribution of the viscoelastic effect of the sensing layers. The motion resistance of the resonator (*R*_1_) in the Butterworth-van Dyke equivalent circuit for the QCMs with Cu_3_(BTC)_2_ were investigated using a network impedance analyzer^32^. The motional resistance provides information on the dissipative losses for the resonance propagation of the films on the QCMs. The *R*_1_ value of a Cu_3_(BTC)_2_-coated QCM was almost constant at about 10Ω within a temperature range of 0–100°C, indicating there was a small dissipative loss in the Cu_3_(BTC)_2_ layer on the QCM (Fig. S4). The QCMs with stiff Cu_3_(BTC)_2_ layers exhibited a stable oscillation through the good resonance propagation within this temperature range.

The QCMs modified with Cu_3_(BTC)_2_ were set into the temperature-controlled measurement chamber, and the sensing properties were investigated by measuring the frequency changes when the film was exposed to VOC vapors in the chamber^11^. During the exposure phase, the adsorption of the VOC molecules within the MOF layer causes the frequency to decrease. After reaching a constant frequency, the incorporated VOC molecules are released by the supply of pure nitrogen carrier gas. During the desorption process, the frequency of the sensors rises due to the mass decrease in the MOF layer. The frequency changes can be converted into weight changes by using the Sauerbrey equation^33^.

**Figure 2:**
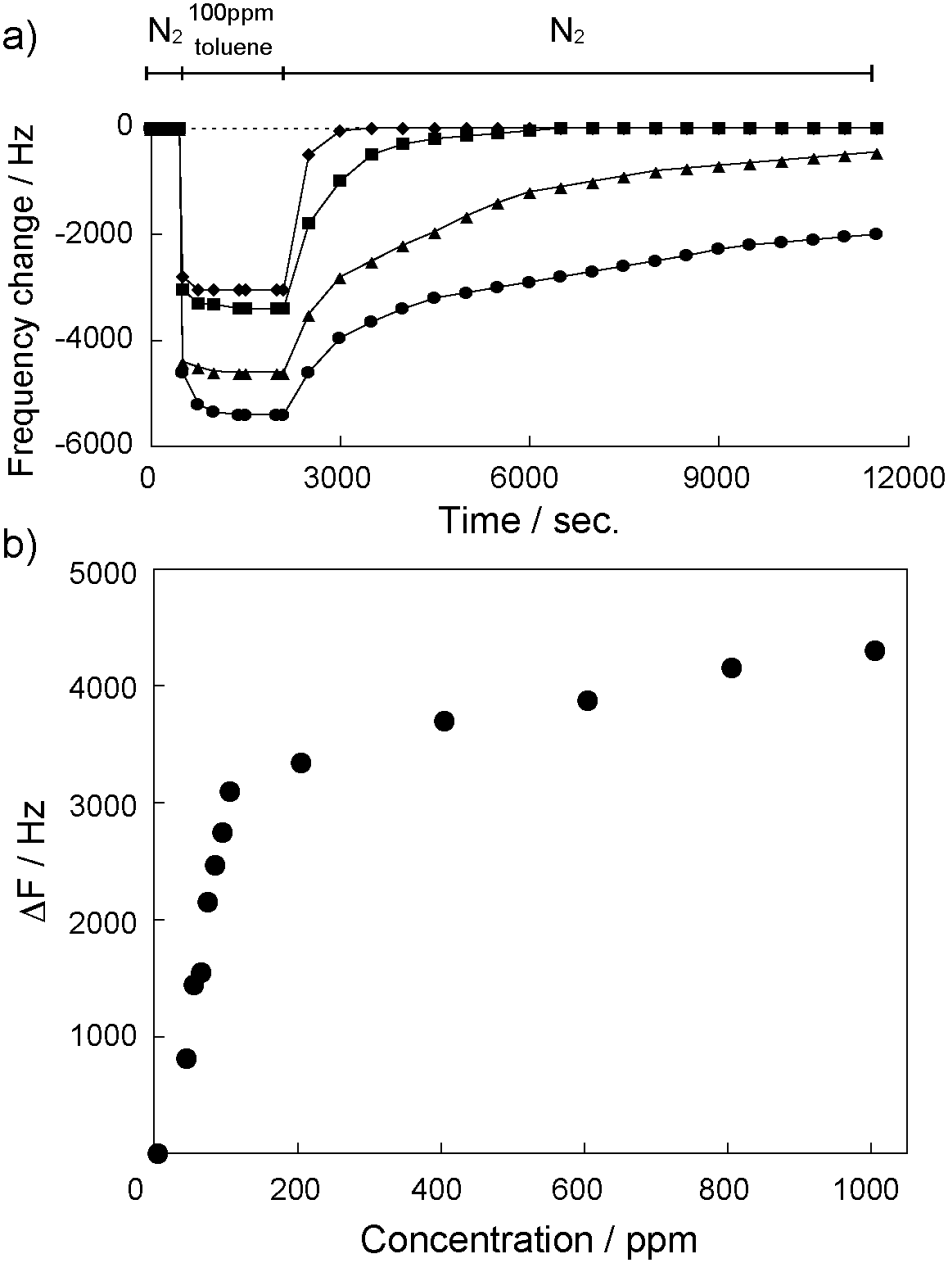
a) Responses of QCM sensors modified with Cu_3_(BTC)_2_ exposure to 100 ppm toluene vapor at 20 (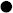), 30 (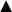), 40 (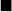), and 60°C (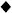). Dotted line is a response of QCM sensor without Cu_3_(BTC)_2_ exposure to 100 ppm toluene vapor at 60°C. b) Frequency change versus toluene concentration at 60°C.

Figure 2a shows the time course of the sensor responses (ΔF) of the QCMs coated with Cu_3_(BTC)_2_ responding to exposure to a 100 ppm toluene vapor at 20, 30, 40 and 60°C. The frequency of the QCM operated at 60°C rapidly decreased in response to the toluene vapor exposure, and the signal stayed constant at *−ΔF* = 3000 Hz after equilibrium was achieved. When the carrier gas changed to pure nitrogen gas, the frequency returned to the initial state within 5 min, which indicates reversible adsorption/desorption processes of toluene. The sensor exhibited a good repeatability for toluene sensing (Fig. S5). Although the maximum responses for a 100 ppm toluene vapor increased with decreasing operation temperature, the desorption process was slower than that at 60°C. The concentration-dependent responses to the toluene vapor are shown in Figure 2b. The response appeared to increase linearly in the concentration range of 0–100 ppm and saturated above 100 ppm, which was followed by the Langmuir sorption model based on the presence of a set number of nanospaces within the Cu_3_(BTC)_2_ layer. A concentration increase of 10 ppm in toluene vapor causes a frequency shift of 90 Hz for the QCM sensor with the Cu_3_(BTC)_2_ layer. The detection limit for the QCM sensor with the Cu_3_(BTC)_2_ layer was evaluated by a concentration change leading to a signal meeting the signal-to-noise ration conventions of the IUPAC (S/N: 3/1)^34^. We found that the detection limit for toluene was about 1 ppm (a noise level is ±1.5 Hz). This detection limit for the Cu_3_(BTC)_2_ layer is good compared to our previously reported values for polymer and nanoparticle-based sensing layers^8^,^9^,^10^,^11^, suggesting that the porous MOF layers play an effective concentrator for the VOC vapors on weight-detectable sensors.

**Figure 3:**
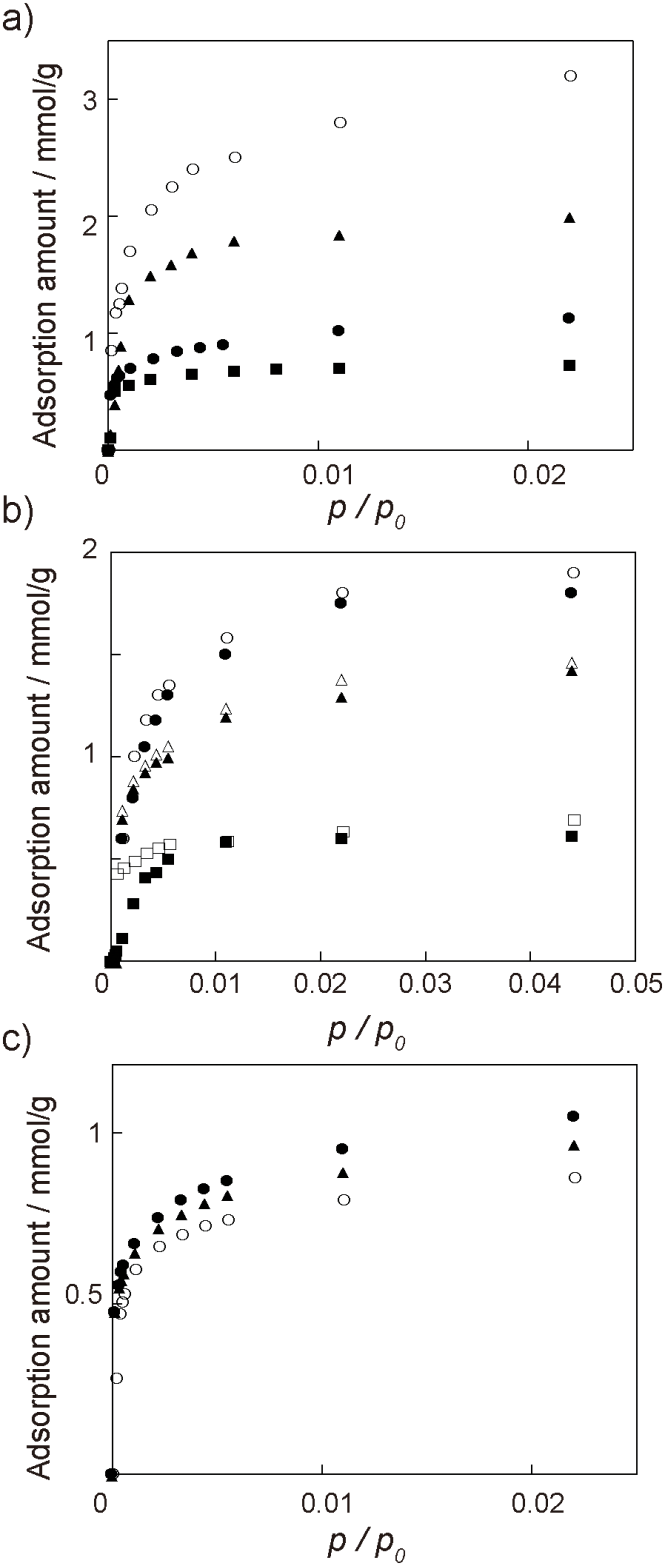
a) Vapor response isotherms of QCM sensors modified with Cu_3_(BTC)_2_ to ethanol (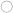), acetone (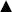), toluene (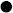), and n-octane (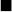) at 60°C. b) Vapor response isotherms to n-hexane (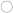), n-haxanol (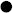), n-heptane (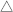), n-heptanol (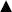), n-octane (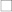), and n-octanol (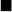) at 60°C. (c) Vapor response isotherms to *o*-xylene (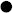), *m*-xylene (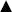), and *p*-xylene (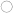) at 60°C.

Figure 3a shows the adsorption isotherms of QCMs coated with Cu_3_(BTC)_2_ for four VOCs recorded at 60°C. The Cu_3_(BTC)_2_-coated QCM isotherms for four VOCs showed a Langmuir-type sorption within this concentration range, and the response sequence was ethanol > acetone > toluene > n-octane. The selectivity of the VOC sensing is caused by the following two factors, the chemical interactions of the VOCs with the MOF internal surface and the molecular sieving effect of the regulated nanospace within the three-dimensional MOF frameworks. Since the internal surface of Cu_3_(BTC)_2_ is hydrophilic due to the presence of two water molecules coordinated at the axial positions of the Cu^2+^-paddlewheels, the hydrophilic surface can interact with any polar VOC vapors such as ethanol and acetone. The regulated pore and aperture sizes in the crystalline MOFs would allow for the size selectivity of the adsorption process of the VOCs. Figure 3b shows the adsorption isotherms of Cu_3_(BTC)_2_-coated QCMs for alkanes and alkyl alcohols possessing different alkyl chain lengths. The isotherm of n-hexanol was almost consistent with that of n-hexane, and the response sequence followed the molecular size of the VOCs. Furthermore, the sensors displayed different isotherms for *o*-xylene, *m*-xylene, and *p*-xylene, implying the recognition of positional isomers of xylene compounds by the Cu_3_(BTC)_2_ layer (Figure 3c). Thus, the Cu_3_(BTC)_2_ layer provides for the selectivity of VOC sensing to the weight-detectable sensors through the size-selective adsorption of the VOCs within the regulated nanospace of the MOFs.

**Figure 4:**
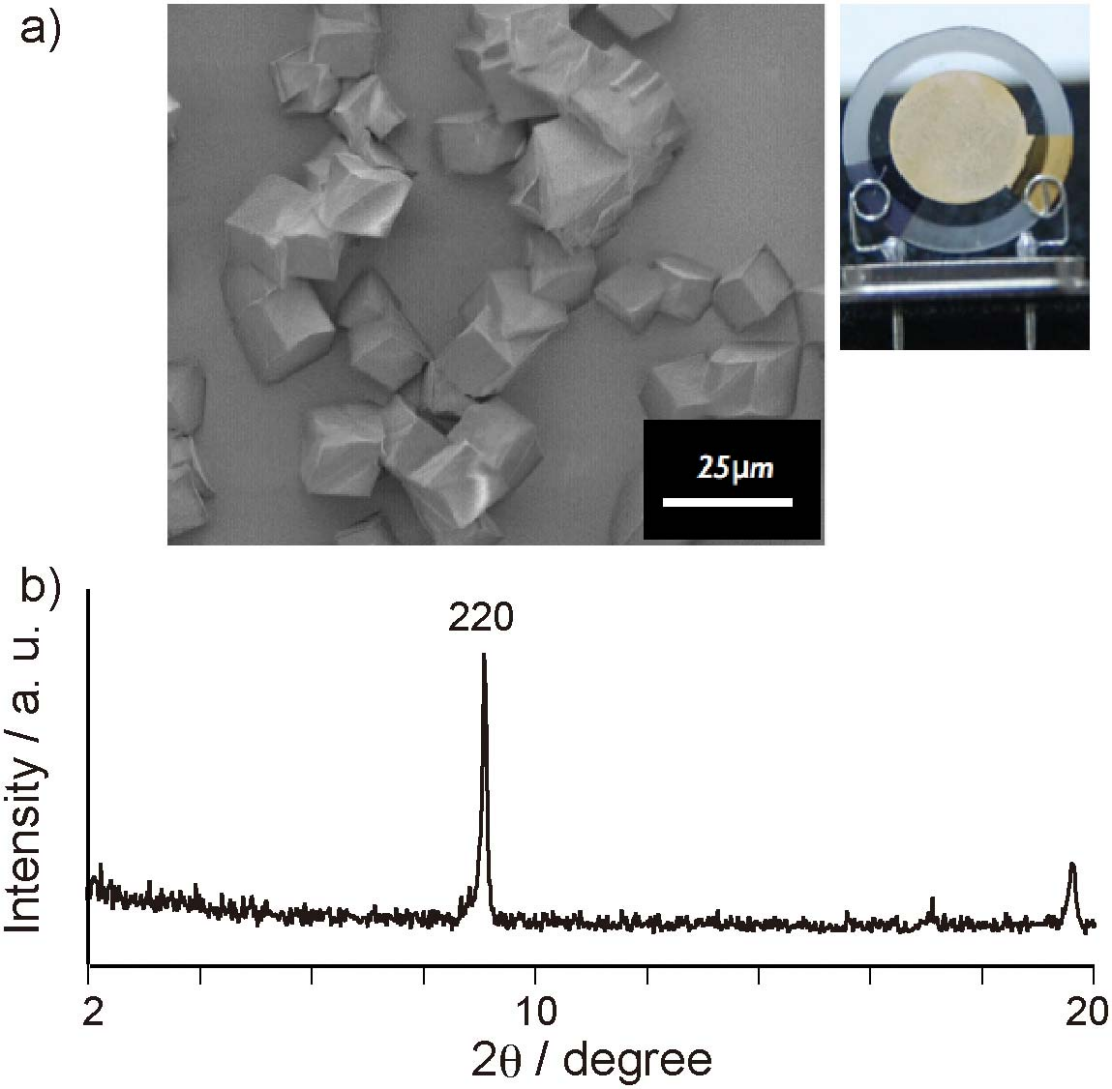
a) SEM image and photograph of Zn_4_O(BDC)_3_ thin film grown from COOH-terminated SAM on gold electrode of QCM. b) Out-of-plane XRD data for Zn_4_O(BDC)_3_ film on QCM.

**Figure 5:**
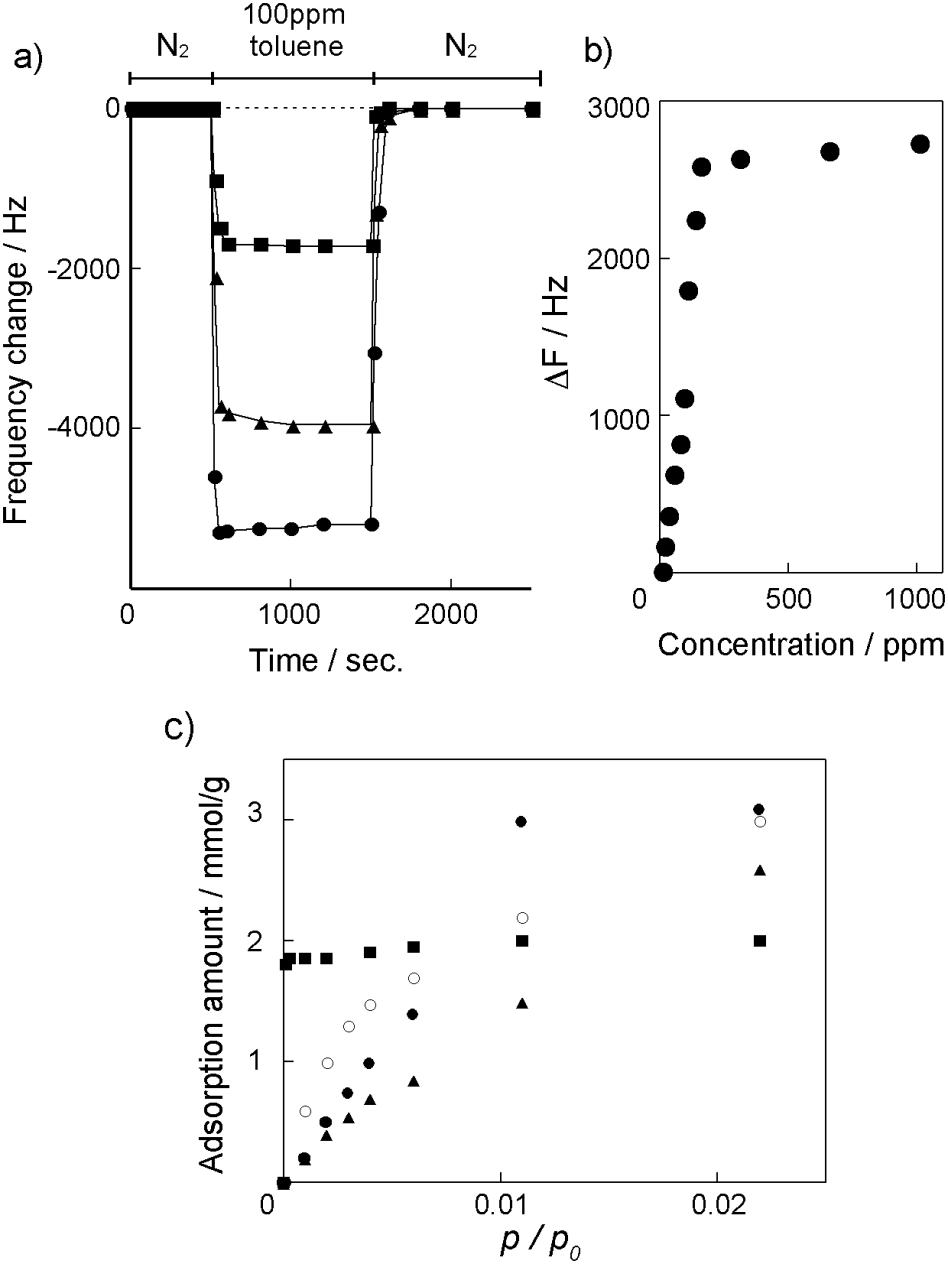
a) Responses of QCM sensors modified with Zn_4_O(BDC)_3_ exposure to 100 ppm toluene vapor at 20 (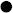), 40 (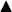), and 60°C (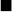). Dotted line is a response of QCM sensor without Zn_4_O(BDC)_3_ exposure to 100 ppm toluene vapor at 60°C. b) Frequency change versus toluene concentration at 60°C. c) Vapor response isotherms of QCM sensors modified with Zn_4_O(BDC)_3_ to ethanol (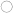), acetone (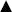), toluene (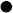), and n-octane (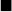) at 60°C.

A thin film of Zn_4_O(BDC)_3_ was also grown from the COOH-terminated SAM onto the gold electrodes of the QCMs by using the crystallization solution. Highly-ordered thin Zn_4_O(BDC)_3_ films were formed on the SAMs as confirmed by SEM and X-ray diffraction pattern (Figure 4a and b). Zn_4_O(BDC)_3_ possesses larger pore size (0.8 nm) and aperture sizes (1.2 and 1.5 nm) than Cu_3_(BTC)_2_ due to a network of zinc oxide tetrahedrals connected with the BTC linkers in the highly-crystalline cubic structure (Fig. S6)^25^. Whereas the Cu_3_(BTC)_2_ layer showed the difference in the response speeds between the adsorption and desorption processes at 20°C, the reversible responses in both processes were observed in the Zn_4_O(BDC)_3_-coated QCMs at 20°C (Figure 5a). The sensitivity of the Zn_4_O(BDC)_3_-coated QCMs was slightly lower than that of the sensor coated with Cu_3_(BTC)_2_ as determined from the slopes of the concentration-dependent responses to the toluene vapor (Figure 5b). While the response sequence of the Zn_4_O(BDC)_3_ layer is the same as that of the Cu_3_(BTC)_2_ layer, the selectivity of the Zn_4_O(BTC)_3_ layer for the VOCs was lower compared to the Cu_3_(BTC)_2_ layer (Figure 5c). The larger pore and aperture sizes in Zn_4_O(BDC)_3_ induced a low molecular sieving effect for the selective sorption of the VOCs. When two MOF thin films were used as the sensing layers of the sensor arrays, the combination of different responses from the two MOF films enables for the detection of the VOC species as well as the determination of the VOC concentration.

**Figure 6:**
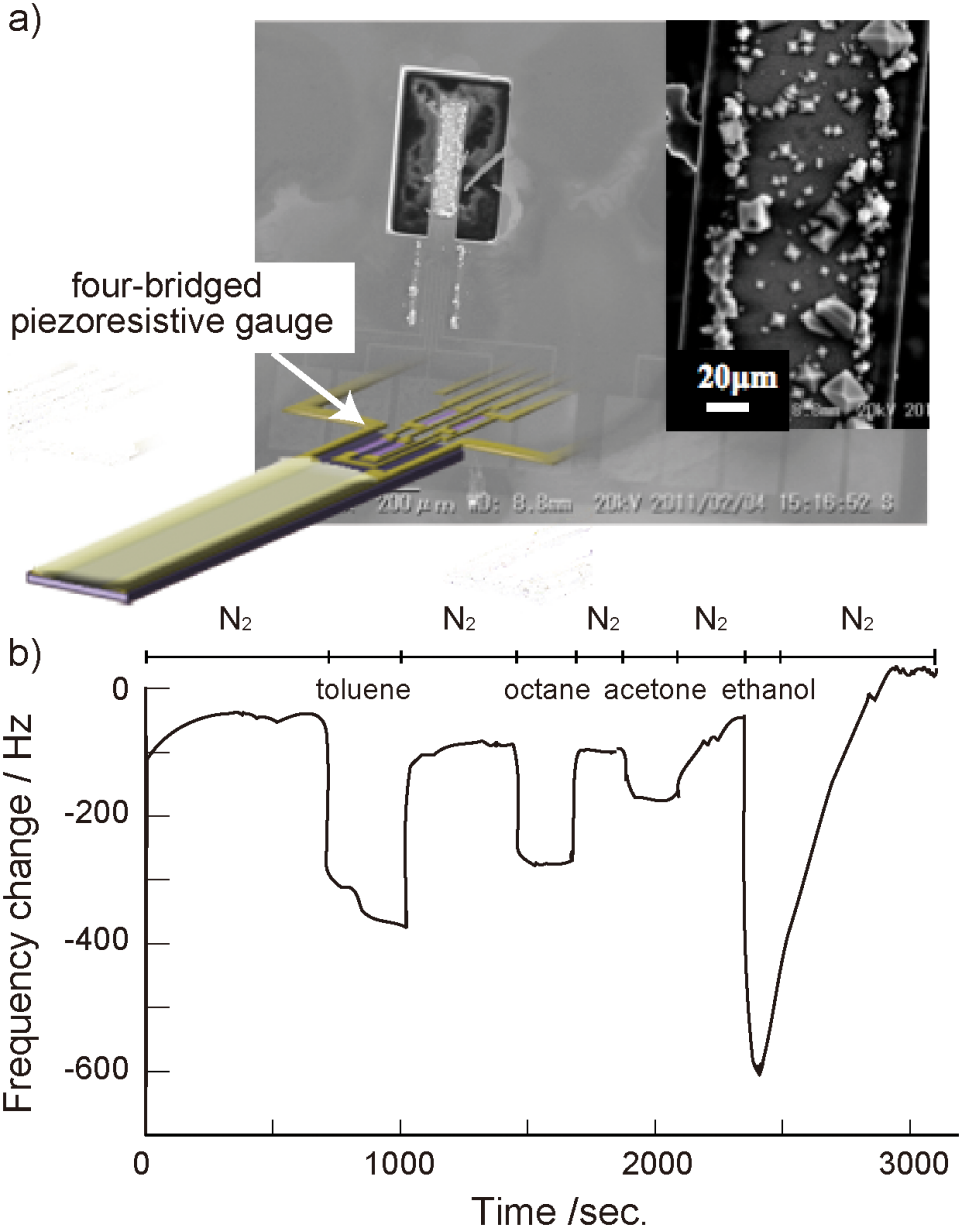
a) SEM images of Cu_3_(BTC)_2_ thin film grown from COOH-terminated SAM on gold electrode of microcantilever resonator. b) Frequency response of Cu_3_(BTC)_2_ film on the microcantilever resonator upon exposure to 100 ppm toluene, n-octane, acetone, and ethanol vapors.

Allendorf et al. demonstrated effective sensing of water and alcohols by using microcantilever modified with a MOF thin film^22^. The adsorption and desorption of the vapors in the MOFs on the cantilever produce strain changes at the interface between the MOF film and the cantilever surface. The resultant stress induces the bending of the cantilever and is detected by a piezoresistive sensor on the microcantilever. We also demonstrated the detection of VOCs by monitoring the oscillation frequency and resistance changes of silicon microcantilever sensor chips coated with TiO_2_ porous films covered with polythiophene layers^11^. The oscillation frequency changes were electrically detected by a set of four-bridged piezoresistive gauges on the sensor chip^35^,^36^. The MOF thin films grown on the microcantilevers can be used to detect VOCs at ppm concentrations. Cu_3_(BTC)_2_ crystals were deposited onto the surface of the cantilevers (Figure 6a). A sensor chip with eight cantilevers coated with Cu_3_(BTC)_2_ was set into a temperature-controlled chamber, and the sensing properties were investigated by measuring the frequency changes when the film was exposed to VOC vapors in the chamber (Fig. S7). Figure 6b shows the responses of a Cu_3_(BTC)_2_-coated microcantilever sensor to 100 ppm VOC vapors operated at 60°C. The resonant frequency changed according to the adsorption and desorption processes of the VOC vapors on the surface of the Cu_3_(BTC)_2_ layer, and the Cu_3_(BTC)_2_ layer on the cantilever displayed an effective selectivity for the VOCs. The frequency rapidly decreased in response to the 100 ppm toluene vapor exposure, and the signal stayed constant at *−ΔF* = 360 Hz. The integration of MOF thin films with a microcantilever sensor platform can detect VOCs through the analyses of the oscillation frequency as a result of the adsorption/desorption of VOCs within the MOFs.

## Discussion

We have demonstrated the detection of VOCs by measuring the frequency changes in the weight-detectable sensors modified with porous MOF layers. The large surface area and regulated nanospace of the MOF layers enhanced their sensitivity and selectivity for VOC sensing. A QCM coated with a Cu_3_(BTC)_2_ layer could detect toluene vapors at a concentration of 1 ppm, and showed different responses to a variety of VOC vapors through the molecular sieving effect of MOFs. Two sensors with Cu_3_(BTC)_2_ and Zn_4_O(BDC)_3_ provided different sensitivities for VOCs. The tunability of the pore and aperture sizes in the MOFs is an advantage for the creation of a pattern recognition library of chemical sensor arrays for electronic nose systems. Weight-detectable sensors coated with MOFs are a promising sensing platform to realize electronic nose systems. Work is underway to fabricate electronic nose systems for the on-site and real-time analyses of VOCs in ambient environments^37^. On-site and real-time analyses of trace VOCs allow us to provide situational awareness for managing the surrounding hazards. The detection of the VOCs generating from our bodies will open up a new possibility for non-invasive and fast health monitoring, such as for cancer, kidney diseases and neurodegenerative diseases^38^,^39^,^40^,^41^.

## Methods

### Chemical Sensing of VOCs by modified QCM and Microcanlilever Sensors

QCM sensors (TamaDevice Co., Ltd., diameter of gold electrode: 5.0 mm) consists of a disk-shaped AT-cut piezoelectronic quartz crystal deposited with gold electrodes on both sides, and are operated at a frequency of 9 MHz. Microcantilever arrays have been fabricated according to the previously reported procedures from single crystal silicon (a dimension of cantilever: 100 μm width, 500 μm length, and 5 μm thickness)^35^,^36^. The deflection of the cantilevers detected electrically by a set of four-bridged piezoresistive gauges (PGs)) and two gold electrodes with a 30 μm distance were deposited on top surface of the cantilevers. The single sensor chip (5 mm × 5 mm) including eight cantilevers was glued on a lead zirconate titanate (PZT) plate of 0.5 μm thick using silver paste to give vibration, and these units were mounted on a 48-pin quad flat ceramic package. The PGs and PZT plate were electrically connected by gold bonding wires (wire diameter is 25 μm) to the package leads. The cantilever was driven at around 420 KHz by the use of feedback oscillation circuit for self-oscillation, which was comprised of signal amplifier, phase controller, band pass filter, and automatic gain controller. This circuit allows us selective oscillation of higher vibration modes of the cantilever by adjusting the band pass filter.

The surface of QCMs and microcantilevers were cleaned by the treatment with O_3_. The cleaned gold electrodes were modified with monolayer of HS(CH_2_)_15_COOH by immersing into a ethanol solution (30 ml) of NanoThinks™ ACID16 (Aldrich, 0.1 ml), and stand for 48 hr at room temperature. The surface modified sensors were repeatedly washed with ethanol and stored in ethanol.

The crystallization solution of Cu_3_(BTC)_2_ was prepared according to the reported method^27^. The SAM-modified QCMs and microcantilevers were held horizontally onto Teflon plate having a window and the gold electrodes were exposed to a clear crystallization solution of Cu_3_(BTC)_2_ (Scheme S1). The Teflon plate was placed in a glass petri dish containing 5 ml dimethyl sulfoxide (DMSO) and the vessel was sealed at 123°C for 6 hr. After 6 hr, the sensors with Cu_3_(BTC)_2_ were washed with CHCl_3_ and dried for 3 hr at 150°C.

The crystallization solution of Zn_4_O(BDC)_3_ was prepared by the following procedure. Zn(NO_3_)_2_ 6H_2_O (78 mg, 0.26 mmol) was added to a 5 ml *N,N*-diethylformamide solution of terephthalic acid (17.5 mg, 0.10 mmol) in sealed glass reactor, and left for 72 hr at 75°C. After 72 hr, the crystals were removed by filtration and obtained a clear crystallization solution of Zn_4_O(BDC)_3_. Zn_4_O(BDC)_3_ crystals were grown on the SAM-modified gold electrodes by the contact with crystallization solution for 8 hr at 60°C. The sensors with Zn_4_O(BDC)_3_ were washed with CHCl_3_ and dried for 3 hr at 150°C.

VOC sensitivities of the sensing layers on QCM, microelectrode, and microcantilever sensors were investigated using a temperature-controlled chamber designed for measuring simultaneously frequency change. Test vapors were generated by the gas calibration unit using ultra-pure nitrogen gas as a carrier gas. Diluted gases are led into the measuring chamber by the computer-driven magnetic valve systems^11^. The frequency changes of sensors were monitored in response to the incorporation of VOCs. Temperature in the measuring chamber was stabilized by a Peltier thermostat to avoid the temperature-dependent frequency changes, and temperature of the chamber was monitored during the experiments. Six universal frequency counters (Agilent Technology, Model 53131A) are able to determine twelve frequencies at the same time. For each test gas, five repeated frequency changes were analyzed for the variation of *−ΔF* to determine reproducibility. The standard deviation in the frequency changes was less than the noise level of QCMs (±1.5 Hz).

## Author Contributions

T.M. conceived the original idea and guided the project. M.K. designed sensing materials and wrote the manuscript. H.Y., S.S. and T.F. carried out material characterization and sensing measurements. T.I. and R.M. carried out design and fabrication of silicon-based microcantilevers. All authors assisted with the manuscript preparation and discussed the results.

## Supplementary Material

Supplementary Information Supplementary information
