# Supplementary material for: Detection of Volatile Organic Compounds by Weight-Detectable Sensors coated with Metal-Organic Frameworks
Source: Sci Rep. 2014 Sep 1;4:6247. doi: 10.1038/srep06247 (PMC4150105; doi:10.1038/srep06247)
Supplement: Supplementary Information [file srep06247-s1.pdf]

Supplementary information

Detection of Volatile Organic Compounds by Weight-Detectable Sensors coated with Metal-Organic Frameworks

Hiroki Yamagiwa, Seiko Sato, Tadashi Fukawa, Tsuyoshi Ikehara, Ryutaro Maeda, and Takashi Mihara and Mutsumi Kimura*

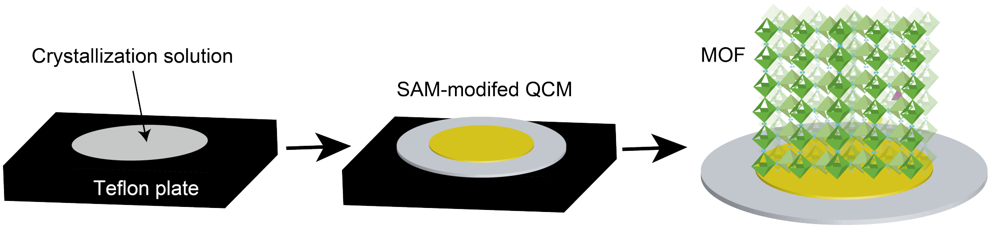

Scheme S1 Set-up of thin film deposition on SAM-modified QCMs

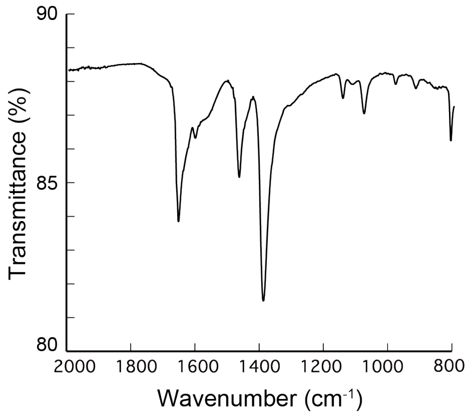

Fig. S1 FT-IR-RAS spectrum of $Cu_3(BTC)_2$ crystal on the QCM

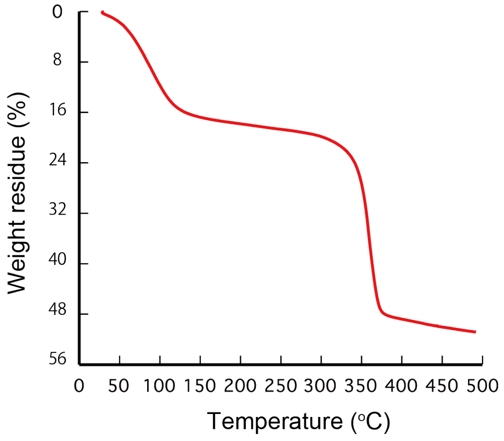

Fig. S2 TGA curve of $Cu_3(BTC)_2$ crystal

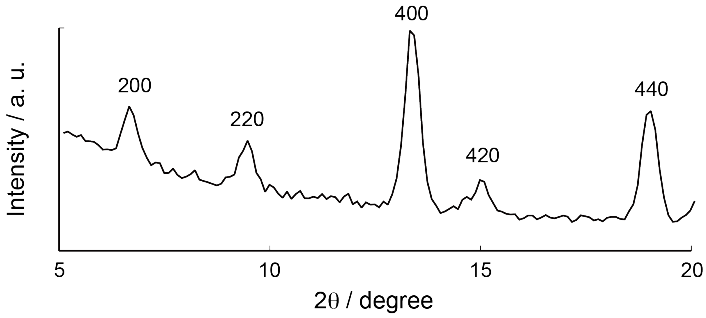

Fig. S3 In-plain XRD data for a $Cu_3(BTC)_2$ on QCM.

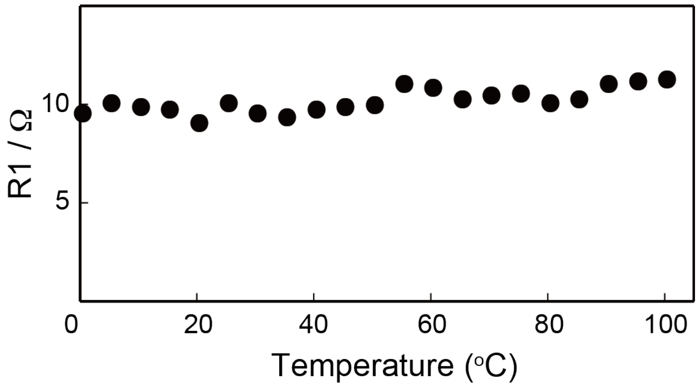

Fig. S4 Motion resistance as function of operating temperature of QCMs coated with $Cu_3(BTC)_2$.

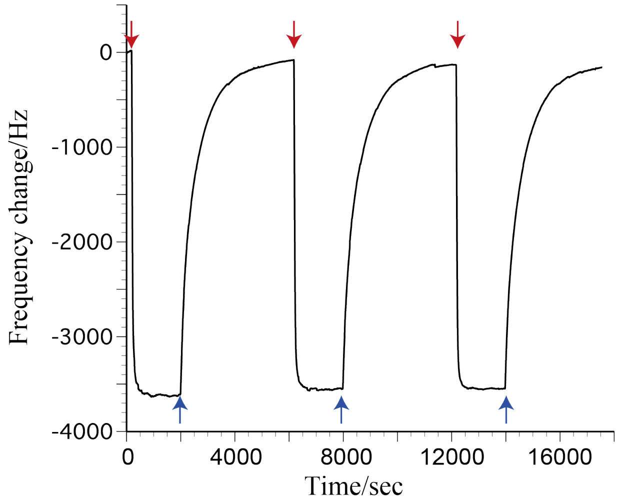

Fig. S5 Responses of the QCM sensor with $Cu_3(BTC)_2$ to repeated dosing with 100 ppm toluene vapor. Red and blue arrows indicate insertion of toluene vapor and pure N_2, respectively.

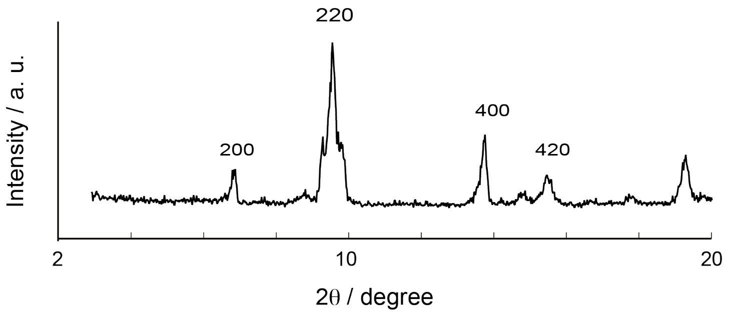

Fig. S6 In-plain XRD data for a $Zn_4O(BDC)_3$ on QCM.

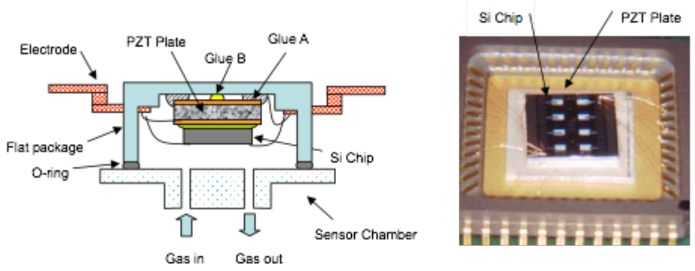

Fig. S7 Temperature-controlled chamber for VOC sensing of microcantilevers modified with $Cu_3(BTC)_2$
